# Effect of exercise training on clinical and physiological variables in adults with myotonic dystrophy type 1: A systematic review protocol

**DOI:** 10.1016/j.mex.2024.102957

**Published:** 2024-09-12

**Authors:** Saidan Shetty, Yuting Luo, Aruna Thomas, Subharup Guha, Donovan Lott

**Affiliations:** a Manipal Academy of Higher Education (MAHE), Manipal-576104, Karnataka, India; b Rehabilitation Science PhD Program, University of Florida, Gainesville, FL, USA; c Department of Physical Therapy, University of Florida, Gainesville, FL, USA; d Department of Biostatistics, University of Florida, Gainesville, FL, USA

**Keywords:** Aerobic training, Strength training, Resistance training, Myotonic dystrophy type 1, Systematic review

## Abstract

Myotonic dystrophy Type 1 (DM1) is a neuromuscular disease characterized by multisystemic involvement including a progressive loss of muscle mass and strength. Further investigation on the effect of exercise in adults with DM1 is needed to incorporate impactful recent findings to better understand the utility of exercise as an intervention. This review aims to summarize and appraise the literature on the effects of aerobic and strength training on clinical and physiological variables in adults with DM1. Six online databases (PubMed, Scopus, Web of Science, CINAHL, EMBASE, and CENTRAL) will be searched using appropriate search terms. Two reviewers will independently screen the relevant studies and extract the data from the selected articles. The methodological quality of the studies included will be assessed using the Joanna Briggs Critical Appraisal checklist. A meta-analysis will be performed if appropriate. This systematic review and meta-analysis will summarize, synthesize, and appraise evidence on the effect of aerobic and strength training on clinical and physiological variables in adults with DM1. The findings of this review will help in clinical decision-making and guide future researchers working with this patient population.

Specifications table

**Table:** 

Subject area:	Medicine and Dentistry
More specific subject area:	Physical Therapy in Neuromuscular Diseases
Name of your protocol:	Effect of Exercise Training on Clinical and Physiological Variables in Adults with Myotonic Dystrophy Type 1: A Systematic Review Protocol
Reagents/tools:	Preferred Reporting Items for Systematic Reviews and Meta-Analyses guidelines to develop the protocol for this systematic review. The Cochrane manual of systematic reviews of interventions will also be consulted.
Experimental design:	Not Applicable
Trial registration:	The protocol of this systematic review was registered in the International prospective register of systematic reviews (PROSPERO) of the National Institute of Health Research available at https://www.crd.york.ac.uk/prospero/ with protocol registration number: CRD42024545119
Ethics:	Not applicable, since this is a systematic review protocol of the reported scientific literature.
Value of the Protocol:	• This protocol is important to provide key information regarding the impact of exercise on patients with myotonic dystrophy type 1 (DM1). • This protocol will be the first to use a systematic review with meta-analysis for this specific topic. • This protocol has the potential to have a positive impact on both future clinical investigations and clinical practice for patients with neuromuscular disease.

## Background

Myotonic dystrophy type 1 (DM1) is a multisystem trinucleotide repeat expansion disorder characterized by the misregulated alternative splicing of critical mRNAs [1,2]. It is the most common adult-onset form of muscular dystrophy with 9.27 cases per 100,000 (ranging from 0.37 to 36.29 cases per 100,000) [3]. DM1 is a multisystemic disease that affects skeletal and cardiac muscle as well as other bodily systems [2,4,5].

The disease is characterized mainly by myopathy or diseased muscles, which include muscle weakness, atrophy, and myotonia (sustained involuntary muscle contraction) that negatively impact the lives of patients with DM1 [4,5]. Those with DM1 reported muscle weakness as their most commonly reported symptom (reported by 94 % of those surveyed) [6], and this muscle weakness often restricts the activities of daily living and social roles for this patient population [7,8]. Adults with DM1 may become physically disabled and have a shortened life span [8,9].

In most patient populations, one of the main strategies to counter muscle wasting and strength loss is through exercise and training. Exercise has beneficial effects on various systems of the body. It stimulates the sympathetic nervous system and induces an integrated response from the body [[10], [11], [12], [13]]. Exercise has proven to be beneficial for improving muscle strength and muscle mass in patients with orthopaedic conditions such as osteoarthritis [14,15], neurological conditions such as spinal cord injury [16,17], and cardiorespiratory conditions such as chronic obstructive pulmonary disease (COPD) [18,19], thereby having a positive impact on overall function and activities of daily living (ADL).

Exercise training (particularly aerobic and strength training) in adults with DM1 has been found to be beneficial in this patient population [[20], [21], [22], [23], [24]]. A scoping review by Roussel et al. (2019) summarized the beneficial effects of exercise training in patients with DM1 [25]. The scoping review included articles published till 2017 and did not perform a methodological quality assessment of the studies included. Therefore, there is no systematic review on the more recent literature nor any meta-analysis for the effect of exercise training on clinical or physiological outcomes in adults with DM1. Knowing the summary, interpretation of outcomes, quality of evidence and synthesis of data on the effect of exercise on various outcomes is important in DM1 for clinicians to be able to have greater knowledge in prescribing the proper dosage and parameters of exercise. Hence, the objective of this review is to search and summarize the literature on the effect of aerobic or strength training on clinical and physiological outcomes in adults with DM1.

## Description of protocol

Research question:

What is the effect of aerobic/ strength/ resistance training on clinical and physiological variables in adults with DM1?

**Objective(s):**

The objective of this review is to search and summarize the literature on the effect of aerobic or strength training on clinical and physiological outcomes in adults with DM1.

**Method**

This systematic review will be written in accordance with the Preferred Reporting Items for Systematic Review and Meta-Analysis Protocols (PRISMA- P) guidelines. The protocol of this review was registered in Prospective Register of Systematic Reviews (PROSPERO) database (CRD42024545119).

### Eligibility: inclusion criteria

The inclusion criteria will be as follows: 1) study design: randomized controlled trials (RCTs)/ quasi-experimental studies/ longitudinal cohort studies; 2) population: adults (≥18 years) with DM1; 3) intervention: aerobic training/ resistance training/ strength training; 4. comparator: no training/ regular training/ alternative training; 5) outcomes: Clinical outcomes include strength, endurance/ aerobic capacity, fatigue, walking performance, mobility, quality of life, psychological outcomes, and sleep; Physiological outcomes include muscle size, mitochondrial changes, vascular changes, muscle pathophysiology, molecular changes, and blood-related outcomes.

### Eligibility: exclusion criteria

Study designs such as case series, case reports, case-control studies, cross-sectional studies, retrospective studies, chart reviews, letters to the editor, commentaries, conference proceedings, systematic reviews, or meta-analysis will be excluded. Studies that include individuals with neuromuscular diseases other than DM1 will be excluded. Grey literature was not searched in specific databases for this review and if found, it will be potentially excluded.

### Data sources and search strategy

A literature search will be executed in 6 databases namely, PubMed, Scopus, Web of Science, Cumulated Index to Nursing and Allied Health Literature (CINAHL), EMBASE, and the Cochrane Central Register of Controlled Trials (CENTRAL) by two reviewers (SS and YL). The search terms will be as follows: “exercise*”, “aerobic”, “strength*”, “resistance”, “training*”, “myotonic dystrophy”, “myotonic dystrophy 1”, “myotonic dystrophy type-1”, “myotonic dystrophy type 1”, “DM1”, and “DM-1”. Search terms will be combined using Boolean operators according to the databases. The search will be filtered according to title, abstract and/or keywords. When applicable the filters of English language, human and journal will be used. Articles published in peer-reviewed journals in the past 20 years (2003–2023) will be included. In case of discrepancies between the first two reviewers, a third reviewer (DL) will resolve the conflicts.

### Study selection

After the search, the citations will be imported to Rayyan software, which is a web-based application for managing data for systematic reviews. Following de-duplication of articles, two reviewers (SS, YL) will independently perform title and abstract screening followed by full-text screening. Any disagreements between the two reviewers (SS, YL) will be resolved by the third reviewer (DL). The study selection process results will be reported in the Preferred Reporting Items for Systematic Reviews and Meta-analyses (PRISMA) flow diagram.

### Assessment of risk of bias or methodological quality

Two reviewers (SS, YL) will independently assess the methodological quality of the included RCTs using the Joanna Briggs Institute (JBI) critical appraisal tool for assessment of risk of bias for RCTs [26] and non-randomized experimental studies using JBI checklist for quasi-experimental studies (non- randomized experimental studies). JBI critical appraisal tool is a methodological quality assessment tool to identify and grade the way in which the study addressed the likelihood of bias in its design, methodology, and analysis [26,27]. The critical appraisal scores will be interpreted by calculating the percentage of “yes” answered to the questions. It will be categorized as “low risk of bias” if the score is greater than or equal to 70 %, “moderate risk of bias” if the score is between 50 % to 69 %, and “high risk of bias” if the score is less than or equal to 49 % [28].

### Data extraction

Two reviewers (SS, YL) will independently extract the data from the included studies using a structured and validated data extraction sheet. The data extraction sheet will be piloted on 3 studies prior to using it for all the articles to be included in the systematic review. This is to standardize the data extraction sheet before administration in the final review and to ensure that all relevant information is being extracted from each of the included studies. The extracted data will be verified by two other authors (DL, AT) to avoid missing relevant information. The data extraction sheet will contain two tables. Table 1 includes information about the study and demographic characteristics such as the author, year of publication, title, journal, country, study design (RCTs/Quasi-experimental studies/Longitudinal cohort studies), study objectives, population, age, sex (female/male/both), weight, height, body mass index (BMI), CTG repeats, sample size of the intervention and control groups, lost to attrition and sample size included in the final analysis.

**Table 1:** Study information and demographics.

**Author**	**Year of publication**	**Title**	**Journal**	**Country**	**Study design (Randomized Controlled Trials/ Quasi-experimental Studies/ Longitudinal Cohort studies)**	**Study objective(s)**	**Population**	**Age (years) [Mean ± SD and/or Range]**	**Gender (female/male/ both)**	**Weight (kg) [Mean ± SD and/or Range]**	**Height (cm) [Mean ± SD and/or Range]**	**Body Mass Index (in kg/m2) [Mean ± SD and/or Range]**	**CTG repeat [Mean ± SD and/or Range]**	**Sample size (n) intervention group**	**Sample size (n)** **Control group**	**Lost to attrition**	**Sample size included for final analysis**

Standard deviation = SD.

Cytosine-thymine-guanine = CTG.

Table 2 will have details of interventions such as type, frequency, intensity, and duration of intervention; setting for intervention; supervised or unsupervised; intervention provider; adverse events (if any); comparator group information; clinical and/or physiological outcomes; instruments/ equipment used; statistical analysis methods; key results; strengths of the study, as mentioned by the author; and, as per the reviewer, limitations, as mentioned by the author and as per the reviewer, future recommendations, as suggested by the author and other remarks. Any discrepancies between the data extraction between the two reviewers (SS, YL) will be resolved by consultation with the third reviewer (DL).

**Table 2:** Methods, results, and impressions by authors and reviewers.

**Intervention**																	
**Type**	**Frequency**	**Intensity**	**Duration**	**Setting**	**Supervised (yes/ No/ Unspecified)**	**Provider**	**Adverse Events**	**Comparator Group Intervention**	**Clinical/ Physio Outcome Measures**	**Instruments/ Equipment used**	**SA Method**	**Key Results (Mean, SD, p values, effect size etc.)**	**Strengths of the Study (mentioned by Author)**	**Strengths of RD and Manuscript according to Reviewer**	**Limita-tions by the Author**	**Future Recs**	**Other Remarks**

Physiological = Physio.

Statistical analysis = SA.

Standard deviation = SD.

Research Design = RD.

Recommendations = Recs.

### Statistical analysis

Averages and standard deviations of the data will be extracted from the prioritized outcomes of the studies. If a study reports standard errors of the mean (SEMs), the standard deviations will be calculated by using the SEMs and the square root of the sample size. Standardized mean differences and 95 % confidence intervals (CIs) will be evaluated to combine the results of the studies. Inconsistency of the results across studies will be determined using the chi-squared test, with a p-value <0.05 judged statistically significant after consideration of the I-squared statistic. Study heterogeneity will be reported as low (I-squared <50 %) or high (I-squared at least 50 %). Depending on the inconsistency of the studies’ results, a fixed effects or random effects model will be used, and the 95 % CI from this analysis will be reported.

### Additional information

To the best of our knowledge, this will be the first systematic review and meta-analysis which will comprehensively summarize and appraise evidence related to the effect of aerobic or strength training on various clinical and physiological variables in adults with DM1. Subsequent analysis of the details of the intervention with respect to frequency, intensity, time, and type of training and its effect on the clinical and physiological outcomes will help suggest appropriate dosage of aerobic and strength training in adults with DM1. This review will include articles published in the past 20 years in six relevant databases. To avoid missing important articles, we will include RCTs, quasi-experimental studies and longitudinal cohort studies.

The possibility of a meta-analysis will be explored and will be conducted if applicable. A critical appraisal of the included studies will signify the quality of evidence and direct points to be considered for future research. We aim to report the strengths and limitations of the included studies, identify gaps in the literature and highlight the potential areas for further research. The results of this systematic review and meta-analysis will guide the clinical decision-making process of clinicians and health-care professionals treating adults with DM1 as well as provide insights for future clinical investigations focused on optimizing exercise to benefit those with DM1. Healthcare providers will be able to target specific clinical and physiological outcomes through aerobic and strength training in the adults with DM1, thereby benefiting the patient population and facilitating their prognosis. Basic and clinical scientists will have the literature summarized to expedite the identification and addressing of future needs in this important area of research. This systematic review has been registered in PROSPERO. Any amendments to the review protocol and review progress will be updated in PROSPERO. After completion of this review, the final manuscript will be submitted to a peer-reviewed journal for consideration for publication.

**Protocol validation**

## Limitations

None.

## CRediT author statement

The final manuscript was approved by all the authors.

**Saidan Shetty:** Conceptualization, Methodology, Data curation, Writing – original draft. **Yuting Luo:** Data curation, Methodology, Writing – review & editing. **Aruna Thomas** Methodology, Writing – review & editing. **Subharup Guha** Formal analysis, Writing – review & editing. **Donovan Lott** Conceptualization, Methodology, Validation, Supervision, Writing – review & editing.

## Declarations of competing interest

The authors declare that they have no known competing financial interests or personal relationships that could have appeared to influence the work reported in this paper.

## Data Availability

No data was used for the research described in the article.

## References

[1] Cho D.H., Tapscott S.J. (2007). Myotonic dystrophy: emerging mechanisms for DM1 and DM2. Biochimica et Biophysica Acta (BBA) - Molecul. Basis Dis..

[2] López-Martínez A., Soblechero-Martín P., de-la-Puente-Ovejero L., Nogales-Gadea G., Arechavala-Gomeza V. (2020). An overview of alternative splicing defects implicated in myotonic dystrophy type I. Gene. (Basel).

[3] Liao Q., Zhang Y., He J., Huang K. (2022). Global prevalence of myotonic dystrophy: an updated systematic review and meta-analysis. Neuroepidemiology.

[4] LoRusso S., Weiner B., Arnold W.D. (2018). Myotonic dystrophies: targeting therapies for multisystem disease. Neurotherapeutics.

[5] Mercuri E., Muntoni F. (2013). Muscular dystrophies. Lancet.

[6] Hagerman K.A., Howe S.J., Heatwole C.R. (2019). The myotonic dystrophy experience: a North American cross-sectional study. Muscl. Nerve.

[7] Raymond K., Levasseur M., Mathieu J., Gagnon C. (2019). Progressive decline in daily and social activities: a 9-year longitudinal study of participation in myotonic dystrophy type 1. Arch. Phys. Med. Rehabil..

[8] Kierkegaard M., Harms-Ringdahl K., Holmqvist L.W., Tollbäck A. (2011). Functioning and disability in adults with myotonic dystrophy type 1. Disabil. Rehabil..

[9] Gagnon C., Mathieu J., Jean S. (2008). Predictors of disrupted social participation in myotonic dystrophy type 1. Arch. Phys. Med. Rehabil..

[10] Nagamatsu L.S., Flicker L., Kramer A.F. (2014). Exercise is medicine, for the body and the brain. Br. J. Sport. Med..

[11] Hautala A.J., Kiviniemi A.M., Tulppo M.P. (2009). Individual responses to aerobic exercise: the role of the autonomic nervous system. Neurosci. Biobehav. Rev..

[12] Daniela M., Catalina L., Ilie O., Paula M., Daniel-Andrei I., Ioana B (2022). Effects of exercise training on the autonomic nervous system with a focus on anti-inflammatory and antioxidants effects. Antioxidants.

[13] Radak Z., Chung H.Y., Goto S. (2008). Systemic adaptation to oxidative challenge induced by regular exercise. Free Radic. Biol. Med..

[14] Bartholdy C., Juhl C., Christensen R., Lund H., Zhang W., Henriksen M. (2017). The role of muscle strengthening in exercise therapy for knee osteoarthritis: a systematic review and meta-regression analysis of randomized trials. Semin. Arthrit. Rheum..

[15] Zacharias A., Green R.A., Semciw A.I., Kingsley M.I.C., Pizzari T. (2014). Efficacy of rehabilitation programs for improving muscle strength in people with hip or knee osteoarthritis: a systematic review with meta-analysis. Osteoarthrit. Cartil..

[16] Bochkezanian V., Raymond J., de Oliveira C.Q., Davis G.M. (2015). Can combined aerobic and muscle strength training improve aerobic fitness, muscle strength, function and quality of life in people with spinal cord injury? A systematic review. Spinal Cord..

[17] Eitivipart A.C., de Oliveira C.Q., Arora M., Middleton J., Davis G.M. (2019). Overview of systematic reviews of aerobic fitness and muscle strength training after spinal cord injury. J. Neurotrauma.

[18] O'Shea S.D., Taylor N.F., Paratz J.D (2009). Progressive resistance exercise improves muscle strength and may improve elements of performance of daily activities for people with COPD. Chest.

[19] O'Shea S.D., Taylor N.F., Paratz J (2004). Peripheral muscle strength training in COPD. Chest.

[20] Ravel-Chapuis A., Duchesne E., Jasmin B.J. (2022). Pharmacological and exercise-induced activation of AMPK as emerging therapies for myotonic dystrophy type 1 patients. J. Physiol..

[21] Mikhail A.I., Nagy P.L., Manta K. (2022). Aerobic exercise elicits clinical adaptations in myotonic dystrophy type 1 patients independently of pathophysiological changes. J. Clin. Investigat..

[22] Allen J., Astin R., Smith C., Banks D., Turner C. (2020). Expiratory muscle strength training improves measures of pressure generation and cough strength in a patient with myotonic dystrophy type 1. Neuromuscul. Disord..

[23] Roussel M.P., Hébert L.J., Duchesne E. (2020). Strength-training effectively alleviates skeletal muscle impairments in myotonic dystrophy type 1. Neuromuscul. Disord..

[24] Sharp L., Cox D.C., Cooper T.A. (2019). Endurance exercise leads to beneficial molecular and physiological effects in a mouse model of myotonic dystrophy type 1. Muscl. Nerve.

[25] Roussel M.P., Morin M., Gagnon C., Duchesne E. (2019). What is known about the effects of exercise or training to reduce skeletal muscle impairments of patients with myotonic dystrophy type 1? A scoping review. BMC Musculoskelet. Disord..

[26] Barker T.H., Stone J.C., Sears K. (2023). The revised JBI critical appraisal tool for the assessment of risk of bias for randomized controlled trials. JBI Evid. Synth..

[27] (2020). Chapter 3: systematic reviews of effectiveness. *JBI Manual Evid. Synthes*. JBI.

[28] Sponchiado Junior E.C., Vieira W de A., Normando A.G.C. (2021). Calcium silicate-based sealers do not reduce the risk and intensity of postoperative pain after root canal treatment when compared with epoxy resin-based sealers: a systematic review and meta-analysis. Eur. J. Dent..

